# Behavioral characterization of triple-hit schizophrenia-like Lisket rats derived from the Long Evans strain through acute and chronic behavioral tests

**DOI:** 10.3389/fpsyt.2025.1601714

**Published:** 2025-09-01

**Authors:** Anna Zoldi, László Kormoczi, Szonja B. Plesz, Leatitia G. Adlan, Gabriella Kekesi, Péter Liszli, Laszló G. Nyúl, Gábor Braunitzer, Gyöngyi Horvath

**Affiliations:** ^1^ Department of Physiology, Albert Szent-Györgyi Medical School, University of Szeged, Szeged, Hungary; ^2^ Department of Image Processing and Computer Graphics, Faculty of Science and Informatics, University of Szeged, Szeged, Hungary; ^3^ Sztárai Institute, University of Tokaj, Sárospatak, Hungary

**Keywords:** impulsivity, homecage system, learning, Long Evans rats, schizophrenia

## Abstract

**Background:**

Automated homecage systems provide valuable insights into rodent behavior in an undisturbed environment over extended periods. This study aims to identify behavioral differences between Long Evans (LE) rats (control) and a novel triple-hit schizophrenia model (Lisket), developed through selective breeding based on schizophrenia-related behavioral alterations following juvenile social isolation and ketamine treatment.

**Methods:**

Pain sensitivity (tail-flick test), behavioral activity, and cognitive function were assessed in acute tests (Ambitus test) and chronic conditions (HomeManner system with a delay discount paradigm).

**Results:**

Lisket rats exhibited significantly decreased pain sensitivity, reduced locomotion and exploration, and impaired learning ability. While all LE rats learned to prefer the large-dose reward tray, only 69% of Lisket rats demonstrated this preference. Although Lisket rats displayed significant cognitive deficits, particularly under delay conditions, no clear signs of heightened impulsivity were detected. Personalized analysis revealed substantial interindividual variability in both groups, accompanied by high intraindividual fluctuations across different parameters.

**Conclusions:**

This study provides the first comprehensive behavioral characterization of the Lisket model, a triple-hit schizophrenia-like rat strain derived from Long Evans rats, under both acute and chronic testing conditions. The automated, experimenter-free approach used in this study offers a promising tool for complex behavioral assessment. Furthermore, the findings emphasize the importance of individualized behavioral analysis alongside group-level assessments to enhance the translational validity of preclinical neuropsychiatric research.

## 1 Introduction

Schizophrenia is a severe, chronic mental disorder characterized by positive and negative symptoms, as well as cognitive impairments, affecting an individual’s thoughts, emotions, and behaviors. Reward-based learning processes also show impairments in these patients, which can be related to both the positive and negative symptoms (1). Furthermore, decreased pain sensitivity is often observed in patients with schizophrenia and in animal models of the disease (2–5). While the exact mechanism of this altered pain sensitivity remains unclear, the involvement of the opioid and cannabinoid systems has been suggested (6, 7). Neuroscience remains one of the most challenging fields in animal modeling due to the complexity of the human brain, which far exceeds that of laboratory animals. Furthermore, some preclinical models fail to account for the chronic nature of neuropsychiatric disorders and the significant role of gene-environment interactions in their etiology and symptomatology. It is widely acknowledged that no single symptom observed in animal studies is specific to schizophrenia. However, the combined behavioral pattern, along with the construct and predictive validity of the model, determines its translational potential.

To develop an animal model with high construct validity, a multiple-hit rat substrain, termed Wisket, was previously created in our laboratory from the Wistar strain. This model combined environmental factors (post-weaning social isolation), pharmacological interventions (subchronic administration of the NMDA receptor antagonist ketamine), and genetic selection based on behavioral phenotypes (8–10). The Wisket model has been extensively characterized, revealing various behavioral and receptor alterations, and its predictive validity has also been confirmed (8, 11–13).

However, findings from acute and chronic behavioral tests demonstrated that the extremely low activity levels of both Wistar and Wisket rats reduced the model’s reliability.

Long Evans (LE) rats display higher locomotor activity and cognitive function than Wistar rats (14–16). Studies using single-hit schizophrenia models have demonstrated that this strain can reliably reproduce certain schizophrenia-like traits, including impairments in cognitive functions such as prepulse inhibition (17, 18). To improve construct validity while overcoming the limitations observed in the Wisket model, we applied the same treatment protocol and selective breeding strategy to LE rats, resulting in a new substrain named Lisket. Lisket animals, similar to Wiskets, showed impaired pain sensitivity, and cognitive function obtained in the reward-based Ambitus test (see below) (19).

Acute behavioral test procedures often elevate stress levels, potentially distorting results or complicating prolonged assessments (20, 21). Automated homecage systems enable the continuous observation of spontaneous behavior under minimally disturbed conditions, thereby improving test reproducibility and providing a more natural setting for the animals (22). Several homecage activity monitoring systems, including PhenoTyper, PhenoMaster, IntelliCage, and PhenoWorld, allow for extended behavioral assessments (23–28). Our research team developed a novel system called HomeManner (HM), a large and enriched homecage specifically designed for the delay discount paradigm (29). This system has been successfully used to investigate prolonged behavioral activity and cognitive function in Wistar and Wisket rats.

Impulsivity—or a lack of self-control in decision-making processes—is a frequently seen symptom of schizophrenia (30). The delay discount task, in which subjects choose between a large-delayed and small-immediate reward, has been often used to assess impulsivity in schizophrenia models (31–33). However, most of the studies used acute test conditions (34–37). Our team previously applied this paradigm in the HM system in Wistar and Wisket rats (29). However, some animals had to be excluded from the analysis due to lack of activity at the food trays over a 13-day period.

The present study aimed to characterize the behavior of Lisket and control LE rats in acute assessments of heat pain sensitivity (tail-flick test) and cognitive function (Ambitus test), as well as chronic behavioral monitoring using the HomeManner system over an extended period. We hypothesized that our test conditions would validate the translational utility of the Lisket model for schizophrenia research by revealing significant behavioral impairments in both short-term and long-term paradigms.

## 2 Methods

### 2.1 Animals

The Hungarian Ethical Committee for Animal Research (RN: XIV/1248/2018 and XIV/1421/2023), in accordance with EU Directive 2010/63/EU, approved all experimental procedures. The study adhered to the guidelines outlined in the Animal Research: Reporting of *In Vivo* Experiments (ARRIVE 2.0).

Regarding the selective breeding process, we followed the same protocol as in our previous studies with Wistar and Wisket rats (8, 10, 38). Control and Lisket rats (both male and female) were weaned at three weeks of age. Lisket rats were then housed individually for four weeks and received intraperitoneal ketamine injections (30 mg/kg, 4 mL/kg) for five consecutive days, starting in the second week of isolation (12). Afterward, the animals were rehoused in groups of two to three per cage. Control (LE) rats were group-housed and received no treatment. At three months of age, Lisket rats exhibiting impaired behavioral performance in the tail-flick and Ambitus tests (see below) were selected for breeding (38). To minimize inbreeding, sibling mating was avoided. Typically, one male was paired with two females (resulting in six males and twelve females per generation) for a two-week mating period, after which females were housed individually to ensure undisturbed parturition. Animals from 8–11 generations were involved in the study. Control (LE, n=12) and Lisket (n=13) male rats were maintained under a 12-hour light/dark cycle at a controlled temperature of 22 ± 1°C.

### 2.2 Tail-flick test

**Figure 1:**
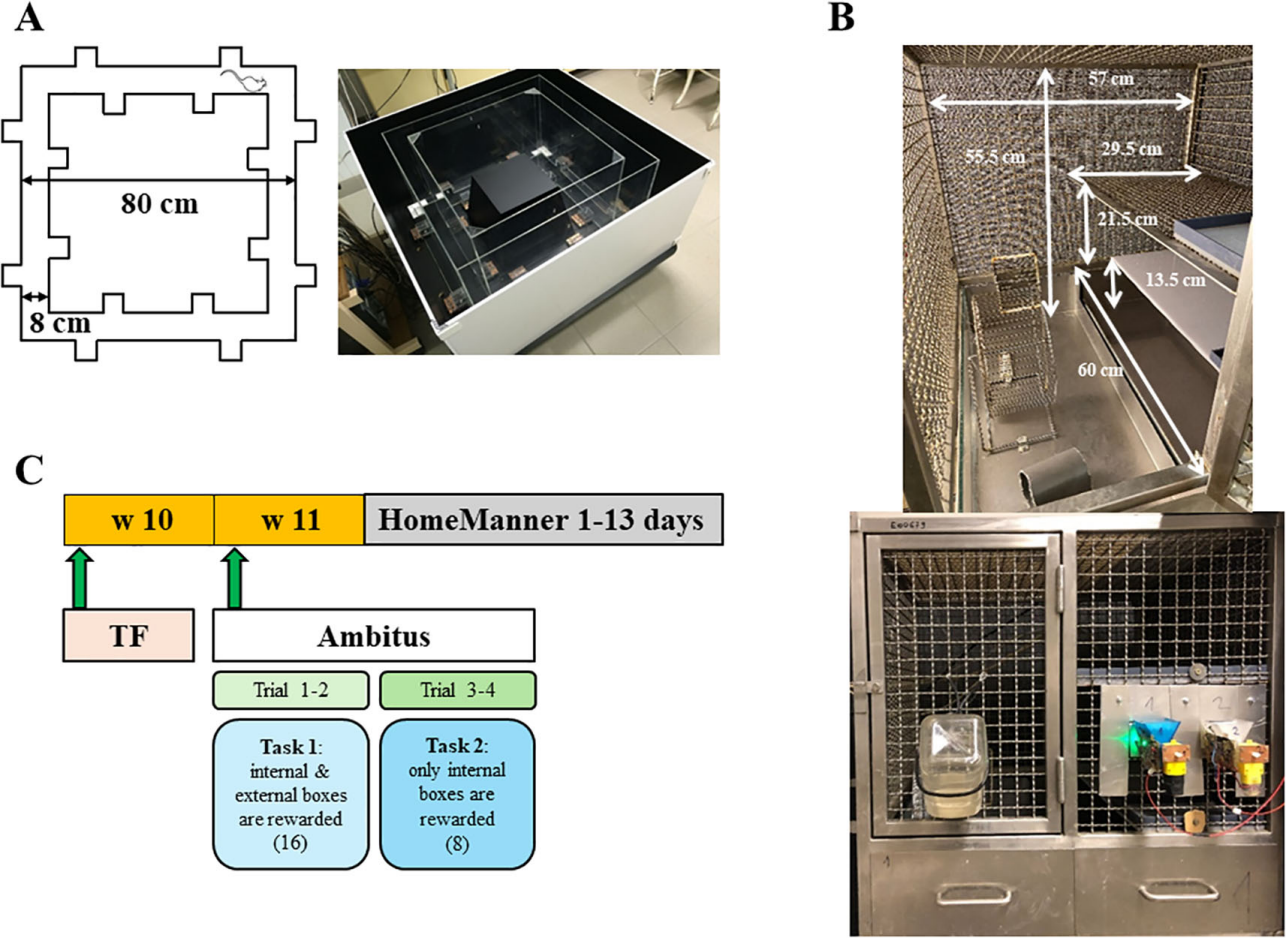
Structure of the Ambitus apparatus **(A)** and the HomeManner system **(B)**. A schematic representation of the experimental paradigm **(C)**.

On week 10, the acute nociceptive threshold was assessed using the tail-flick (TF) test, as previously described (**Figure 1B**) (38). The reaction time was measured by immersing the distal 5 cm of the tail in 48 °C hot water until a tail-withdrawal response occurred, with a cut-off time of 40 seconds. Since body weight influences nociceptive responses, TF latencies were normalized to body weight and expressed as relative TF (RTF) latencies for statistical analysis (39).

### 2.3 Ambitus test

The Ambitus apparatus combines features of the reward-based Hole Board and maze corridor tests (8). It is a rectangular corridor constructed of clear plexiglas with a black floor (**Figure 1A**; www.deakdelta.hu). Each corridor contains four side boxes (16 in total) for food rewards (puffed rice, 20 mg). Infrared beams detect exploratory activity at each side box and locomotor activity in the middle of the corridor with a 1 ms time resolution. After placing the food rewards, trials began by positioning the rats at the starting point (**Figure 1A**), after which the experimenter immediately left the room. The animals were given 300 seconds to explore the corridor and collect the rewards. The apparatus was cleaned with 70% ethanol between animals. The Ambitus system allows for the detection and calculation of multiple behavioral parameters, including locomotor activity, exploratory behavior, and reward collection (eating), as well as cognition-related measures such as effective exploration, adequate exploration ratios, and learning capacity. Definitions, abbreviations, and calculations for these parameters are provided in **Table 1**.

**Table 1:** Summary of the measured and derived behavioral parameters with units and definitions.

Tests	NR	Parameter	Unit	Definition/Calculation
TF	0	RTF: relative tail-flick latency	Ratio	(Tail withdrawal latency x 100)/(body weight).
Ambitus	1	Locomotor activity (LOCO)	N	Number of corridor entries within 5 minutes.
	2	Exploratory behavior (EXPL_TOT_A)	N	Total box visits within 5 minutes.
	3	External visits (EXPL_E)	N	Subset exploration categorized as external box visits up to collection of the rewards. Number of external box exploration × 300)/Eating time (s).
	4	Internal visits (EXPL_I)	N	Subset of exploration categorized as internal box visits up to collection of the rewards. Number of internal box exploration × 300)/Eating time (s).
	5	Effective Exploration Ratio (E_E)	Ratio	The ratio of collected rewards and explored boxes. (Eating count)/(Number of box visits)
	6	Adequate exploration ratio (A_E)	%	(number of collected food rewards)x100/(number of explorations up to task completion time)
	7	Learning capacity (L_C_A)	%	A ratio indicating the animal’s capacity to collect rewards. (Eating count × 300 × 100)/(Number of rewards × Eating time).
HM	8	Delay time	s	Interval between stimulus trigger and food delivery at LD side.
	9	Latency (LAT)	min	Latency of the 1^st^ daily exploration
	10	Eating time (EAT_T)	%	Time taken to consume all pellets, capped at 24 hours (86,400 s). (Time up to collection of rewards)*100/(total time; cut-off: about 24 h)
	11	Eating activity ratio (EAT_R)	%	[(Eated count)x100]/(number of rewards)
	12	Epoch (Epoch_TOT)	s	Hourly duration of activity at the trays if the break shorter than 5 minute
	13	Epoch (Epoch_BEF)	s	Hourly duration of epochs up to collection of rewards. (Duration of epochs × 3600)/(Eating time).
	14	Cycles	N	Number of cycles within one epoch
	15	Exploration (EXPL_TOT_HM)	N	(Total exploration events × 3600)/(86,400 s).
	16	Exploration (EXPL_BEF)	N	(Total exploration events × 3600)/Eating time (s).
	17	Learning capacity (LC_HM)	%	A ratio indicating the animal’s capacity to collect rewards. (Eating count × 86,400 × 100)/(Number of rewards × Eating time (s)).
	18	Adequate exploration (E1)	%	Eat_N+trigger number within one cycle.
	19	Anticipatory restlessness (E2)	N	(Exploration number during the food delivering)/(cycle number)
	20	Premature exploration (E3)	N	(Exploration number during ITI)/(cycle number)
	21	Incorrect exploration (E4)	N	(Exploration number during delay)/(cycle number)

ITI, interstimulus interval (20 s). N, number.

### 2.4 HomeManner system

The HomeManner (HM) apparatus, developed in the authors’ laboratory, consists of six sets of operant cages housed in a separate room under standard conditions, with a house light positioned outside the cages (**Figure 1B**). Each cage measures 57 × 60 × 55.5 cm and has three levels, with steel wire grid forming the sidewalls and top (**Figure 1B**). Since a structured cage environment may be more beneficial for rodents than a large open floor area (40), the cage was divided into two sections: one side with a single floor and the other with three floors. The animals could access all floors by climbing the grid walls. The single-story section included a playing area designed to enhance well-being and sensory-motor stimulation. This area was equipped with a running wheel (for voluntary exercise), an abacus, and a plastic tube for environmental enrichment. At the front of the cage, a water bottle was provided for free access to drinking water, which could be reached from the playing area (**Figure 1B**). The first level of the three-floor section functioned as a shelter (28.5 × 60 × 11 cm), containing bedding material (nest box: sawdust, 3–4 cm thick), where animals could also hide marbles. The second and third levels, positioned above the shelter, were made of opaque plastic and steel grid, respectively. On the second floor, 20 cm from the water bottle, two food dispensers were attached to each cage. The food dispensers consisted of reward containers positioned outside the cage, each equipped with a small electric motor that delivered food pellets (45 mg, F0021, Bio-Serv™ Dustless Precision Pellets™ Purified Rodent Diet, BioServ, Frenchtown, NJ, US) into two small trays (12 × 20 mm) placed 7 cm apart inside the cage. LED lights were positioned near the containers to indicate reward availability.

The trays were fitted with infrared sensors to detect food delivery, reward consumption, and animal activity at the trays. Any contact with the trays was classified as exploration. The system was connected via an interface to a PC in a separate room, where custom software (developed by P.L.) controlled food delivery and recorded all events at both trays. The collected data were then extracted and analyzed using another software developed by L.K. At the start of the experiment, the pellet dispensers were filled with 12 g and 4 g of pellets for the large-dose (LD) and small-dose (SD) trays, respectively, maintaining these positions throughout the study. When the animals were placed in their cages, the LED lights were switched on, and the dispensers released three pellets on the LD side and one pellet on the SD side, following a paradigm based on a previous study (29, 41). Once the animals consumed the pellets from a tray, the LED light on that side switched off, and no further reward was available for 20 seconds (inter-trial interval, ITI).

After the ITI, the LED light switched back on, and if an exploration at the tray was registered (trigger stimulus), food pellets were dispensed. The rats were not time-restricted for initiating trials, allowing voluntary interaction with the trays throughout the experiment. Even in the absence of a programmed delay, the electric motors required 2–4 seconds to dispense the food pellets.

The trays remained accessible 24 hours a day, but once the animals consumed all the food available for that day (12 g + 4 g from the dispensers), the LED lights switched off until the next morning (between 9 and 10 AM), when the dispensers were refilled. At that point, the trays were rebaited with three pellets (LD) or one pellet (SD), and the LED lights were switched on again. In the event of a hardware malfunction, the LED lights were turned off.

The HM system allows for the detection and analysis of exploratory behavior, reward collection (eating), cognitive performance, and impulsivity-related parameters, including various types of explorations, learning capacity, and delay time. The definitions, abbreviations, and calculations of these parameters are detailed in **Table 1**. Activity at the trays was characterized by the mean hourly duration of epochs, the mean number of cycles within one epoch, and the number of different types of explorations within one cycle (E1–E4). An epoch was defined as an active phase that began with a trigger stimulus and ended if the animal showed no activity at the tray for more than five minutes (300 seconds). One epoch could contain multiple cycles. Within an epoch, the first cycle started with a trigger stimulus and ended with the next LED_ON event. Subsequent cycles began with LED_ON and ended with the following LED_ON event.

Four different types of exploration were differentiated within one cycle. Adequate explorations (E1) were defined as the sum of the trigger stimulus (1) and the collection of rewards (1 or 3 pellets at SD or LD, respectively), making them standard at both trays. E2 captured anticipatory restlessness during the food delivery phase, encompassing the period marked by the food dispenser’s motor noise up to the onset of consumption; higher E2 values signified increased restlessness in anticipation of the reward. E3 denoted explorations occurring during the inter-trial interval (ITI), referred to as premature explorations. E4 represented explorations occurring during the delay period, categorized as incorrect explorations.

### 2.5 Experimental paradigm

On week 10, TF latencies were recorded four times at 30-minute intervals (**Figure 1C**). In the following week, the animals participated in the Ambitus test, following the procedure used in previous studies (8, 9). Food restriction was applied 48 hours before the test, while water remained freely available to ensure adequate motivation. Two task types were used: Task 1 (Trials 1–2), in which all boxes contained rewards, was conducted in the morning, while Task 2 (Trials 3–4), in which only the inside boxes were baited, was performed three hours later (**Figure 1C**).

At least one week after the Ambitus test, the animals were transferred to the testing room, where they underwent two days of food restriction to maintain motivation for consuming the rewards in the HM. The animals were assigned to one of six cages in a pseudo-randomized manner and housed individually without visual contact for 13 days. Both groups were equally represented in the five experimental rounds required to obtain 15 animals per group. However, three control and two Lisket animals were excluded due to sensor system malfunctions in their cages over several days, resulting in a final dataset comprising 12 LE and 13 Lisket animals.

On Day 1 of the experiment, the animals were placed in the large cages between 9:00 and 10:00 AM without access to standard food, but both dispensers were filled with pellets (12 + 4 = 16 g). From the following day onward, standard food (10 g/day, which is about 40–50% of the daily required food amount) was provided on the first floor near the pellet-delivering trays. Thus, a moderate food restriction was maintained throughout the experiment in HM, but the required daily intake could still be achieved by consuming pellets from the dispensers.

During the training phase (Days 1–3), no delay was applied. In the testing phase (Days 4–13), a 10-second delay was introduced on the LD side for animals that had learned to prefer LD over SD rewards (with a preference at least 10% above random). The delay was gradually increased by 10 seconds each day, depending on the animal’s preference, following the method applied in previous studies (29, 35). Apart from the introduction of the delay, no parameters were modified throughout the experiment. Body weight was measured at the beginning and end of the experiment in the HM. Fluid consumption was recorded twice a week, with fresh water provided each time, and relative fluid consumption was calculated based on body weight. Pellet consumption was monitored daily, and the dispensers were refilled accordingly. Standard food consumption was assessed at the end of the experiment.

To minimize disturbance, the experimenter generally entered the room only once per day, except in cases of equipment malfunction. During these visits, the experimenter checked the functionality of the equipment, inspected the health of the animals, refilled the dispensers, provided standard food, and replaced the drinking water. While the HomeManner cages were not cleaned at all over the 13 days, only one animal was housed in each large cage (about 0.2 m³), and no extreme dirtiness was observed at the end of the study.

### 2.6 Measurements and statistical analyses

The Ambitus and HM systems recorded various behavioral parameters related to exploratory activity and reward collection (**Table 1**). In order to compare the results of these different parameters on a common scale—despite differences in units and distributions—we normalized the raw data using z-score transformation. For each parameter, we subtracted the group mean (all animals in all trials or days) from each individual value and divided by the standard deviation. This standardization allows for the direct comparison of effect sizes across variables and test conditions (acute vs. chronic), as visualized in the summary figures. The method is widely used in behavioral neuroscience to unify diverse datasets for integrative analysis (42). Additionally, the coefficient of variation (CV = standard deviation/mean) was calculated to evaluate variability across trials or days.

**Figure 2:**
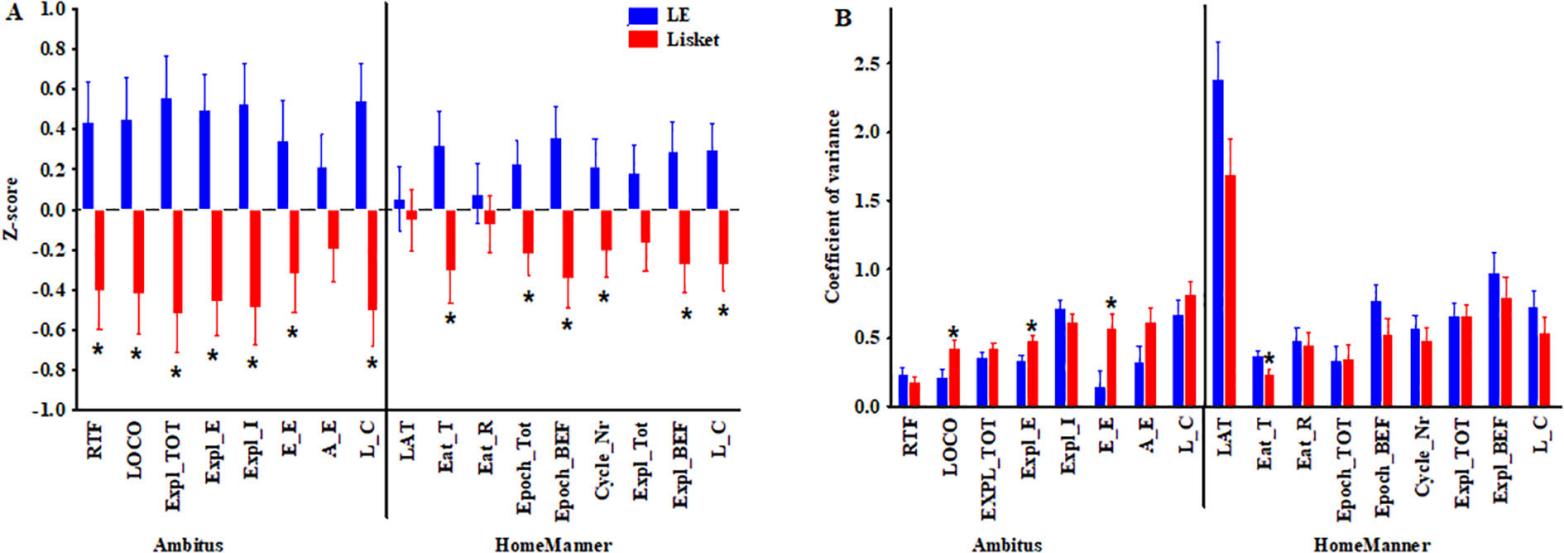
Changes in z-score values for the investigated parameters in the tail-flick test (RTF), Ambitus test, and HomeManner system **(A)**. Changes in covariance values across trials (1–4; TF, Ambitus) or days (1–13; HM) for the investigated parameters **(B)**. Definitions, abbreviations, calculations, and statistical results for the investigated parameters are provided in **Table 1**. The symbol * indicates significant differences (p < 0.05) between groups.

Due to a software malfunction in the HM system, tray activity data from five days were lost (10 data points). Consequently, a total of 640 data points were analyzed across the two trays for the 25 rats over the 13-day period. Since tray activity was largely dependent on the time required to consume all food rewards (defined as eating time), several parameters were converted to the relative number of exploration events (**Table 1**). One-way analysis of variance (ANOVA) was applied to analyze general observations (e.g., body weight and fluid intake), total z-scores, and covariance data, while factorial ANOVA was used for raw data in all tests. In the RTF and Ambitus tests, the factors were group (control vs. Lisket) and trial (Trials 1–4; **Figure 2**), whereas for the HM system, the factors included group, day, and the presence or absence of delay during the testing phase. When the global test was significant, Tukey *post hoc* tests were performed to evaluate the effects of different factors, as shown in the figures. Spearman correlation analysis was conducted to examine associations between the results of the Ambitus and HM tests. To visualize individual variability across all analyzed variables, a complex heatmap was generated using the z-scores from all tests for each animal. All data are expressed as means ± S.E.M., with significance set at p < 0.05. Statistical analyses were performed using STATISTICA 13.5.0.14 (TIBCO Software Inc., USA).

## 3 Results

### 3.1 General observations

Fluid intake did not differ significantly between the two groups, as determined by one-way ANOVA (LE: 79 ± 3.7 ml/kg/day; Lisket: 78 ± 2.8 ml/kg/day). All standard food (10 g/day) was consumed by each animal in the HM. Regarding body weight, factorial ANOVA revealed significant effects of group (F_(1,138)_ = 100.12, p < 0.0001), age (F_(5,138)_ = 340.64, p < 0.0001), and their interaction (F_(5,138)_ = 2.35, p < 0.05). Lisket animals had significantly lower body weight than controls throughout the entire study period (LE: 315 ± 14.1 g; Lisket: 263 ± 11.7 g). However, body weight changes were similar in both groups, with slight weight gain observed by the end of the testing period (LE: 13.0 ± 2.29%; Lisket: 17.3 ± 2.06%).

### 3.2 Tail-flick test

Regarding RTF latency, factorial ANOVA revealed a significant effect of group (**Table 2**), with Lisket rats exhibiting significantly longer RTF latency compared to control animals (LE: 1.9 ± 0.09; Lisket: 2.6 ± 0.13 s/100 g; z-score results shown in **Figure 2A**, left side), indicating lower acute heat pain sensitivity. The coefficient of variation across the four trials was similar in both groups (**Figure 2B**, left side).

**Table 2:** Factorial ANOVA results for the tail-flick and Ambitus test parameters during the four trials.

Parameters	Group	Trial	Gr/Tr
Tail-flick test			
RTF	21.28;(1,92) <0.001		
Ambitus test			
1. LOCO	23.12;(1,92) <0.001	9.69;(3,92) <0.001	NS
2. EXPL_TOT	40.06;(1,92) <0.001	15.32;(3,92) <0.001	NS
3. EXPL_E	28.10;(1,92) <0.001	NS	NS
4. EXPL_I	31.00;(1,92) <0.001	25.86;(3,92) <0.001	3.82;(3,92) <0.05
5 E_E	14.82;(1,92) <0.001	13.68;(3,92) <0.001	NS
6. A_E	5.86;(1,92) <0.05	NS	NS
7. L_C	26.51;(1,92) <0.001	21.11;(3,92) <0.001	NS

For the parameters, see **Table 1**. Numbers within the cells: F value; (Degree of Freedom); P value.

### 3.3 Ambitus test

For most behavioral parameters in the Ambitus test, factorial ANOVA revealed significant effects of group, trial, and their interaction (**Table 2**). Lisket rats exhibited reduced locomotor activity (LOCO), exploratory behavior (EXPL_TOT, EXPL_E, and EXPL_I), and cognition-related measures (A_E, E_E, and L_C) compared to their parent strain, as shown by their z-scores in **Figure 2A**, left side. Additionally, Lisket animals displayed a greater tendency for variance in most parameters, with significant differences observed in locomotion (LOCO), exploratory activity in the external boxes (EXPL_E), and effective exploration (E_E; **Figure 2B**, left side).

### 3.4 HomeManner system

Regarding overall daily activity at the trays, control and Lisket animals performed 12 ± 0.2 epochs, 70 ± 1.2 cycles, and 869 ± 25.5 explorations. Analysis of z-scores over the 13-day investigation period revealed that Lisket rats showed impairments in six out of nine parameters, including eating behavior (EAT_T), various tray activity measures (Epoch_TOT, Epoch_BEF, Cycle number, Expl_BEF), and cognitive performance (L_C) compared to their parent strain, as shown in **Figure 2A**, right side. Additionally, variance was lower across all parameters in Lisket animals, with a significant difference observed for EAT_T (**Figure 2B**, right side). Next, data were analyzed separately for the training phase and the test phase, both with and without delays.

#### 3.4.1 Training phase (Days 1–3)

**Figure 3:**
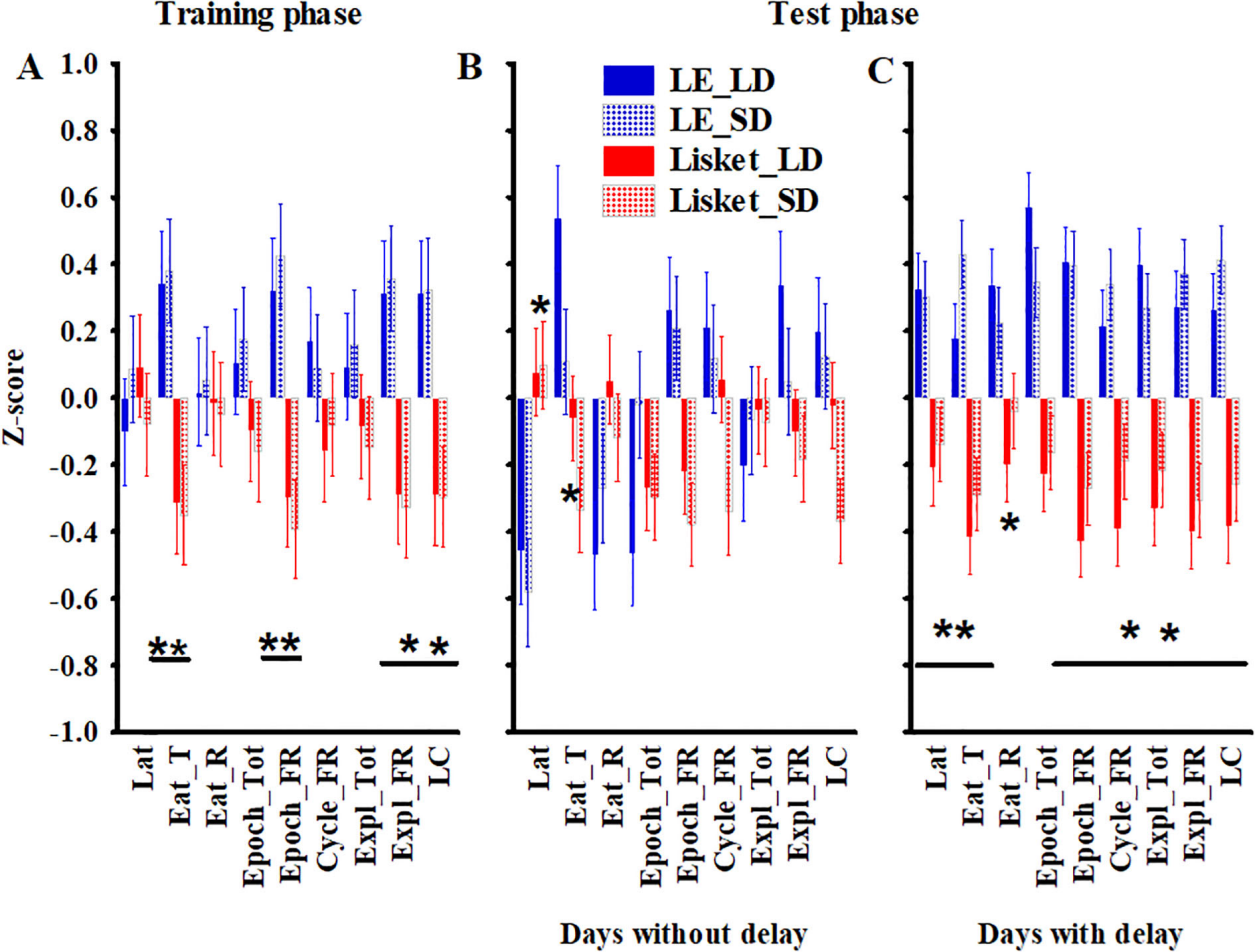
Changes in z-score values for the investigated parameters in the HomeManner system, shown separately for training days **(A)** and testing days without **(B)** or with delays **(C)**. Definitions, abbreviations, calculations, and statistical results for the investigated parameters are provided in **Table 1**. The symbols * and ** indicate significant differences (p < 0.05) between groups at one or both sides (at the lines), respectively.

**Figure 4:**
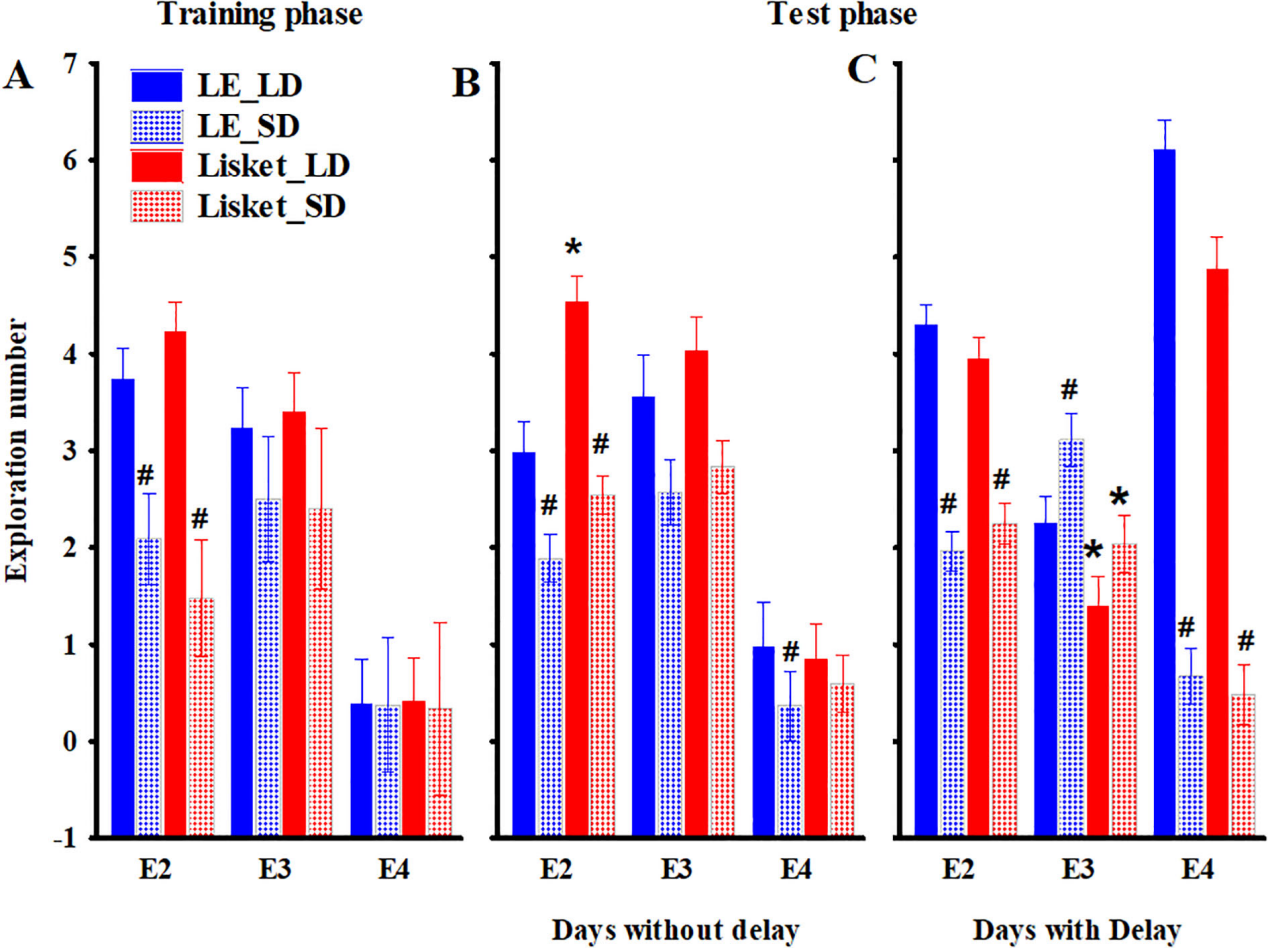
Changes in the different types of explorations (E2–E4) within one cycle in the HomeManner system, shown separately for training days **(A)** and testing days without **(B)** or with delays **(C)**. Definitions, abbreviations, calculations, and statistical results for the investigated parameters are provided in **Tables 1**, **3**, and **4**. The symbols * and # indicate significant differences (p < 0.05) between the two groups and between the two trays (large dose – LD vs. small dose – SD), respectively.

Four of the nine parameters showed significant group differences (**Table 3**, **Figure 3A**), with impairments observed in the Lisket group. These included EAT_T (C: 61 ± 3.7%, Lisket: 82 ± 2.7%), Epoch_BEF (C: 338 ± 40.2 s, Lisket: 158 ± 15.8 s), Expl_BEF (C: 86 ± 10.6, Lisket: 41 ± 4.5), and L_C (C: 177 ± 20.8%, Lisket: 93 ± 9.0%). A significantly higher total number of explorations was recorded on the LD side compared to the SD side (EXPL_TOT LD: 39 ± 3.2, SD: 26 ± 2.4; **Table 3**). No significant interactions were found in any of the parameters, indicating that the pattern of activity differences at the two trays was consistent across both groups. Significant side differences in the types of exploratory activities within one cycle were identified, independent of the group. Specifically, E2 (anticipatory restlessness) was significantly lower at the SD side compared to the LD side (**Table 3**, **Figure 4A**).

**Table 3:** Factorial ANOVA results for behavioral parameters in the training and test phases of the HomeManner test.

Parameters	Training phase		Test phase		
	Group	Side	Group	Side/(day)	Interaction
8. Delay time			5.06;(1,225) <0.05	8.08;(9,225) <0.0001	
9. LAT					
10. Eat_T	20.35;(1,146) <0.0001		48.56;(1,486) <0.0001	8.83;(1,486) <0.005	
11. Eat_R					
12. Epoch_TOT			26.30;(1,486) <0.0001	10.84;(1,486) <0.005	
13. Epoch_BEF	18.36;(1,146) <0.0001		36.12;(1,486) <0.0001		
14. Cycle Nr			24.87;(1,486) <0.0001	8.54;(1,486) <0.005	
15. EXPL_TOT		4.97;(1,146) <0.05	14.51;(1,486) <0.0005	59.27;(1,486) <0.0001	4.94;(1,486) <0.05
16. EXPL_BEF	15.34;(1,146) <0.0005		17.77;(1,486) <0.0001	9.21;(1,486) <0.005	
17. A_E			19.67;(1,486) <0.0001		
18. L_C	14.23;(1,146) <0.0005		33.38;(1,486) <0.0001	13.39;(1,486) <0.0005	4.83;(1,486) <0.05
19 E1		52.71;(1,146) <0.0001	6.15;(1,486) <0.05		
20. E2		20.27;(1,146) <0.0001	6.15;(1,486) <0.05	166.97;(1,486) <0.0001	
21. E3					
22. E4			5.67;(1,486) <0.05	117.02;(1,486) <0.0001	5.09;(1,486) <0.05

For the parameters, see **Table 1**. Numbers within the cells: F value; (Degree of Freedom); P value.

#### 3.4.2 Test phase (Days 4–13)

During the test phase, four Lisket animals did not learn to prefer the LD side throughout the study, preventing the application of delay for these individuals. Among the remaining animals, the mean delay time was similar between the two groups, with a maximum of 30 seconds (LE: 10 ± 1.2 s; Lisket: 11 ± 0.8 s). The analysis indicated that the presence of delay significantly influenced behavior, as factorial analysis revealed not only significant group differences but also significant effects of delay presence or absence, along with interactions involving this factor (**Table 4**). The z-score presentation showed that the main differences between LE and Lisket animals emerged on days with delays, with all parameters indicating significant impairments in Lisket animals, at least on the LD side, suggesting that the introduction of delay played a major role (**Figures 3B, C**). Analysis of the different types of explorations within one cycle (**Table 4**) revealed that E2 (anticipatory restlessness) was significantly higher in Lisket animals at the LD side on days without delay, while lower values were observed at the SD side in both groups (**Table 4**, **Figure 4B**). In the presence of delay, no group differences were detected, but fewer E2 explorations occurred at the SD side compared to the LD side (**Figure 4C**). E3 (premature exploration during ITI) without delay exhibited significant side differences, with lower values at the SD side compared to the LD side (**Figure 4B**). In contrast, when delay was introduced, Lisket animals showed a significantly lower number of E3 explorations at both sides, while LE animals exhibited higher exploratory activity at the SD side compared to the LD side (**Figure 4C**). For E4 (incorrect exploration during delay), no significant group differences were observed. However, significantly fewer E4 explorations were recorded at the SD side compared to the LD side, primarily in the presence of delay (**Figures 4B, C**). Spearman correlation analysis of parameters from the Ambitus and HomeManner systems revealed seven significant positive correlations out of 63: between EXPL_TOT_A and EXPL_TOT_HM (r = 0.40; p < 0.05), E_E_A and EAT_R (r = 0.53; p < 0.01), E_E_A and Epoch_TOT (r = 0.47; p < 0.05), E_E_A and L_C_HM (r = 0.43; p < 0.05), A_E and LAT (r = 0.44; p < 0.05), A_E and EAT_R (r = 0.57; p < 0.005), and L_C_A and EXPL_TOT_HM (r = 0.40; p < 0.05).

**Table 4:** Factorial ANOVA results for behavioral parameters in the test phase of the HomeManner test, analyzed separately for days with and without delay (see **Figures 3**, **4**).

Parameters	Results						
	Group	Delay (D)	Side	GR/D	GR/Side	D/Side	GR/D/Side
9. LAT	7.12;(1,482) <0.01	37.19;(1,482) <0.0001		42.43;(1,482) <0.0001	NS	NS	
10. Eat_T	44.23;(1,482) <0.0001					14.79;(1,482) <0.0005	
11. Eat_R		30.97;(1,482) <0.0001		20.03;(1,482) <0.0001			
12. Epoch_TOT	9.69;(1,482) <0.01	43.73;(1,482) <0.0001	5.62;(1,482) <0.05	18.72;(1,482) <0.0001		4.66;(1,482) <0.05	7.24;(1,482) <0.01
13. Epoch_BEF	32.59;(1,482) <0.0001					4.55;(1,482) <0.05	
14. Cycle Nr	19.51;(1,482) <0.0001					11.22;(1,482) <0.001	
15. EXPL_TOT	5.32;(1,482) <0.05		76.48;(1,482) <0.0001	16.25;(1,482) <0.0001			3.97;(1,482) <0.05
16. EXPL_BEF	19.12;(1,482) <0.0001		19.45;(1,482) <0.0005		4.66;(1,482) <0.05	6.36;(1,482) <0.05	
17. A_E	25.98;(1,482) <0.0001	14.57;(1,482) <0.0001	68.01;(1,482) <0.0001				
18. L_C	23.31;(1,482) <0.0001		7.04;(1,482) <0.01			7.57;(1,482) <0.01	
19. E2	13.1;(1,482) <0.0005		141.89;(1,482) <0.0001		14.32;(1,482) <0.0005		4.96;(1,482) <0.05
20. E3		17.57;(1,482) <0.0001		9.95;(1,482) <0.005		19.15;(1,482) <0.0001	
21. E4		85.01;(1,482) <0.0001	64.68;(1,482) <0.0001			60.80;(1,482) <0.0001	

For the abbreviations, see **Table 1**. Numbers within the cells: F value; (Degree of Freedom); P value.

### 3.5 Individual- level analyses

**Figure 5:**
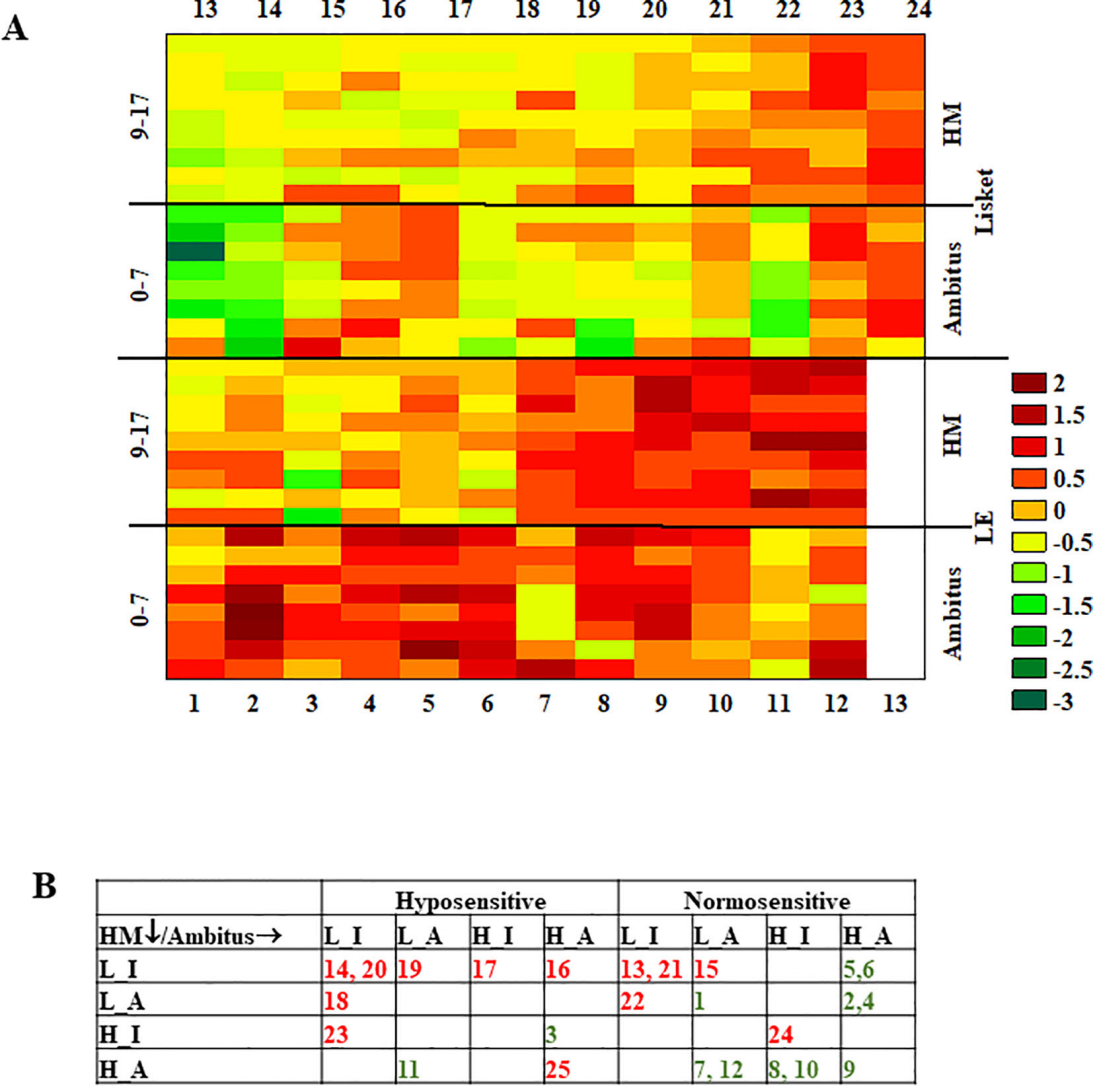
**(A)** Complex heatmap of z-scores for the investigated parameters obtained in the Ambitus and HomeManner tests for both groups. The individual animal numbers are indicated along the lower horizontal axis for the control group (1–12) and the upper horizontal axis for the Lisket group (13–25). Parameters: Tail-flick test: 0. RTF; Ambitus test: 1. LOCO_TOT, 2. EXPL_TOT, 3. EXPL_BEF_E, 4. EXPL_BEF_I, 5. E_E, 6. A_E_A, 7. L_C_A; HomeManner: 8. Delay time, 9. Latency, 10. Eating time (Eat_T), 11. Eat_R, 12. Epoch_TOT, 13. Epoch_BEF, 14. Cycle Nr, 15. Expl_TOT_HM, 16. EXPL_BEF, 17. A_E_HM, 18. L_C_HM. See also **Table 1**. **(B)** Distribution of animals in each category based on their z-score related to cognitive performance (L_C values), behavioral activity (locomotion, epoch duration) in Ambitus and HM tests, and pain sensitivity. Animals with z-scores < 0 for a given parameter were assigned to the low performance (L), inactive (I), and hyposensitive categories, while those with z-scores ≥ 0 were classified as high performance (H), active (A), and normosensitive for L_C, activity, and pain threshold, respectively. Green and red numbers indicate LE and Lisket animals, respectively.

The heatmap visualization of the z-scores revealed that, in addition to group differences, there were high interindividual differences in both groups (**Figure 5**). Furthermore, considerable intraindividual variability was observed across different parameters, with Lisket animals showing a slightly higher coefficient of variance compared to control animals (CV: 0.6 ± 0.12 and 0.9 ± 0.20 for LE and Lisket animals, respectively, p = 0.3). To further examine interindividual and intraindividual differences, animals were categorized based on their z-scores for cognitive performance (L_C: Low performance – L vs. High performance – H), behavioral activity (locomotion and epoch duration: Inactive – I vs. Active – A) in the Ambitus and HM tests, and pain sensitivity (hyposensitive vs. normosensitive). If an animal had a z-score < 0 for a given parameter, it was classified as low performance, inactive, or hyposensitive, whereas a z-score ≥ 0 placed it in the high performance, active, or normosensitive category. This classification resulted in animals being assigned to one of 32 possible behavioral categories, and 13 of 32 remained empty (**Figure 5B**). The diagram clearly showed the high degree of separation between the two groups. Most control LE animals (10 out of 12) were categorized as normosensitive based on pain sensitivity and exhibited active behavior in at least one or both acute and chronic tests (7 out of 10). In terms of cognitive performance, 11 out of 12 LE animals showed high performance in at least one test. However, even animals with a high level of learning capacity exhibited variability in pain sensitivity or behavioral activity (**Figure 5B**). In contrast, most Lisket animals (10 out of 13) were categorized as low performance, with 8 out of 10 showing low performance in both tests. Additionally, most Lisket animals were classified as inactive (7 out of 13) and hyposensitive (8 out of 13). However, some Lisket animals with low learning capacity still exhibited normal pain sensitivity or behavioral activity (**Figure 5B**).

## 4 Discussion

This study examined the behavioral phenotype of the newly developed triple-hit schizophrenia-like (Lisket) rat model at both group and individual levels under different experimental conditions, including acute assessments (tail-flick and Ambitus tests) and prolonged monitoring using the HM system. Consistent with previous findings, Lisket rats exhibited an increased pain threshold and behavioral impairments in acute behavioral tests; these results are in agreement with clinical experience (9, 19, 38, 43). Additionally, analyses from the HM system revealed significant alterations in most parameters in Lisket animals compared to controls. However, these results contrast with previous observations in Wistar and Wisket rats, where the very low activity levels of those strains compromised the reliability of such analyses (29). While the most pronounced differences between Lisket and LE rats emerged on days with delays, no signs of altered impulsivity were detected in Lisket animals compared to controls. Analysis of exploratory behavior within one cycle revealed few differences between groups. Across both groups, all exploration types were more frequent on the LD side on days without delays, suggesting that the animals learned to prefer the tray providing a greater reward, regardless of group. However, this pattern shifted for premature explorations (E3) on days with delay, which became more frequent on the SD side in both groups, indicating that the introduction of delay shifted exploratory dominance to the SD side. Regarding group differences, Lisket rats exhibited more explorations during food delivery (E2) on the LD side on days without delay compared to LE rats, suggesting greater anticipatory restlessness. Additionally, Lisket animals showed fewer premature explorations (E3) on days with delay than controls, which might indicate a weaker positive association with rewards in these animals (44). However, no significant group differences were observed in incorrect explorations (E4).

The few significant relationships observed between the parameters of the Ambitus and HomeManner tests highlight the importance of multiple testing procedures under different conditions in the behavioral phenotyping of model rats.

Continuous monitoring of animal behavior using automated homecage systems can enhance the validity and reliability of behavioral characterization in rodents (24). Consequently, such approaches may improve the translational reliability of animal models for various neuropsychiatric disorders (27, 28, 35, 45–53). Both acute and chronic behavioral assessments provide distinct yet complementary insights into animal behavior. Ideally, both types should be applied to achieve a comprehensive characterization of the phenotype. However, chronic studies require extended periods and cannot be conducted in large cohorts simultaneously. Therefore, acute tests may be more suitable for rapid screening, selective breeding, or short-term pharmacological interventions, while chronic assessments are better suited for in-depth phenotyping and evaluating long-term treatment effects. As demonstrated in this study, each approach contributes uniquely to our understanding, and their combined use enhances the translational value of the model.

Our HM setup is unique in allowing rats to exhibit a continuous, self-initiated feeding pattern to obtain rewards, rather than being restricted to short, task-specific engagements (29). While schizophrenia models are typically developed using Wistar or Sprague Dawley rat strains, we previously established a multiple-hit schizophrenia model based on Wistar rats (8, 38). However, the low general activity of Wistar rats, which was further diminished in the Wisket substrain, limited the reliable evaluation of behavioral activity. LE rats display higher locomotor activity and cognitive function than Wistar rats (14–16), and studies using single-hit schizophrenia models have shown that this strain can replicate certain schizophrenia-like traits (54–57). To improve construct validity, we applied the same selective breeding paradigm to LE rats as previously used for Wistars, resulting in the development of the Lisket model. Unlike in the Wistar/Wisket groups, where low activity levels hindered behavioral assessments, none of the Lisket animals were inactive, allowing for a more reliable evaluation of behavior (19, 29). This study demonstrated that Lisket rats exhibited behavioral impairments in both tests, alongside decreased pain sensitivity.

The variability observed in repeated testing also revealed some interesting phenomena. Surprisingly, the covariance values for most parameters were higher in the Ambitus test for Lisket animals compared to controls, whereas the opposite trend was observed in the HM system (**Figure 2B**). This suggests that the higher stress levels experienced during the four trials of the Ambitus test may have contributed to this effect. In contrast, the lower coefficient of variation (CV) over the 13-day period in the HM system for the Lisket group may indicate more stable behavior over an extended timeframe.

The delay discount paradigm is frequently used to assess impulsivity in animals; however, this test is typically conducted in a new environment, which can induce acute stress (approximately one hour per day) (58). Koot et al. (35) described a method for testing delay discounting in the home cage (35), but the animals were housed in standard rat cages with limited movement space, where they underwent two short sessions (one hour each) per day, separated by eight hours. Behavioral testing in a larger cage with environmental enrichment over an extended period can provide a more ecologically relevant assessment under lower-stress conditions, increasing validity (22, 25). A recent study investigated impulsivity in Wistar rats using an automated CombiCage setup, which consisted of two conventional type III Plexiglas cages connected by a PVC tunnel (59). The study found that rats learned to prefer the larger reward, though this preference was disrupted by psychostimulants. In contrast, no significant signs of impulsivity were detected in Lisket animals, as evidenced by their similar delay tolerance and exploration patterns compared to controls. However, four out of 13 Lisket animals (31%) failed to develop a preference for the LD side, and most parameters related to activity and cognition were impaired in this substrain within the HM system. This suggests that Lisket animals exhibit learning deficits over prolonged periods, particularly on days with delays, where the introduction of delay caused greater disturbances in Lisket rats than in controls. The discrepancy between these findings and earlier studies may stem from differences in cage size and strain characteristics (35, 59).

Individuality is a fundamental characteristic of living beings and has become an increasingly important factor in the diagnosis and treatment of schizophrenia. The high level of interindividual behavioral variability may stem from differences in age, sex, genetic background, cognitive functions, and environmental factors (20, 60). In preclinical studies, homogeneity and low variability are often prioritized to ensure statistically robust findings; however, this approach does not adequately reflect the complex clinical patterns observed in schizophrenia. Personalized data analyses can shift the focus from group-level patterns to individual outcomes, potentially enhancing the translational utility of animal studies (61–64). Our results demonstrated that each animal exhibited a unique behavioral profile, with even similar parameters (e.g., learning ability) varying between acute and chronic tests. The heatmap visualization not only illustrated group differences but also highlighted substantial interindividual variability, particularly among Lisket animals across most parameters. Some Lisket animals performed comparably to controls, whereas others exhibited marked impairments. Additionally, the heatmap revealed high intraindividual variance in different parameters within the same animal (**Figure 5B**). For instance, some animals with high cognitive performance (high L_C score) displayed variability in other measures, such as pain sensitivity and activity-related behaviors, each of which may have distinct effects (65). In conclusion, while the group-based analysis showed significant differences between the control and Lisket animals, the intricate individualized analysis revealed high level of inter-individual differences in the behavioral phenotypes. This is in agreement with the clinical data, since schizophrenia, as a heterogeneous disorder, is characterized by numerous symptoms that vary between the patients.

Although the HM system is currently well-suited for behavioral investigations, certain limitations must be considered for further improvement. The device is designed for single-housed rats, which restricts the number of simultaneous observations and may introduce a form of social stress that could influence behavior (66). However, previous research suggests that individual housing of adult rats has only a minor impact on stress-related parameters, including cognitive function, and that environmental enrichment in the large homecage may help mitigate potential negative effects (67). Without video recording analysis, the HM system is currently limited to assessing exploratory, eating, and impulsive behaviors in rats. While none of these observed behaviors are specific to schizophrenia, the complex behavioral phenotype demonstrated by Lisket rats, combined with the high level of construct validity, may offer new insights into the face validity of this triple-hit schizophrenia-like model. Another possible explanation for the decreased eating activity in Lisket rats could be reduced appetite, which is accompanied by lower body weight in this group; however, further studies are needed to investigate the hedonic behavior of these animals. In addition, a more detailed assessment of impulsivity might require more sophisticated methods, such as the Five-Choice Serial Reaction Time Task in these animals. Additionally, the high degree of individual variability must be taken into account. As in clinical practice, personalized characterization is likely to play an increasingly important role in preclinical research. It is well known that behavioral studies require a larger number of animals, although the long-term HomeManner study generates a large amount of data. Therefore, it cannot be excluded that a higher number of animals would reduce inter-animal variability. Further integration of video-based image analysis will be essential for detailed behavioral characterization of rodents (both rats and mice), along with refinements to the homecage system and validation of additional behavioral paradigms for future studies.

## 5 Conclusion

Our Lisket rat model is the first triple-hit schizophrenia model derived from the Long Evans (LE) rat strain to be characterized using both acute and chronic behavioral tests. While acute assessments, such as heat pain and reward-based Ambitus tests, offer high-throughput results in a short time, prolonged undisturbed observations provide valuable insights into additional behavioral characteristics. The automated, experimenter-free approach described in this study presents a promising method for investigating complex behaviors, particularly in conjunction with pharmacological interventions that require multiple and long-term testing procedures. Additionally, this study emphasizes the importance of personalized data evaluation alongside group-level analyses, as an individualized approach can enhance the translational utility of preclinical schizophrenia research.

## Data Availability

The raw data supporting the conclusions of this article will be made available by the authors, without undue reservation.

## References

[1] Leroy A Amad A D’Hondt F Pins D Jaafari N Thomas P. Reward anticipation in schizophrenia: A coordinate-based meta-analysis. Schizophr Res. (2020) 218:2–6. doi: 10.1016/j.schres.2019.12.041, PMID: 31948895

[2] Becker A Grecksch G Schroder H. Pain sensitivity is altered in animals after subchronic ketamine treatment. Psychopharmacology. (2006) 189:237–47. doi: 10.1007/s00213-006-0557-2, PMID: 17016710

[3] Dworkin RH. Pain insensitivity in schizophrenia: A neglected phenomenon and some implications. Schizophr Bull. (1994) 20:235–48. doi: 10.1093/schbul/20.2.235, PMID: 8085127

[4] Jochum T Letzsch A Greiner W Wagner G Sauer H Bar KJ. Influence of antipsychotic medication on pain perception in schizophrenia. Psychiatry Res. (2006) 142:151–6. doi: 10.1016/j.psychres.2005.09.004, PMID: 16631931

[5] Tuboly G Benedek G Horvath G. Selective disturbance of pain sensitivity after social isolation. Physiol Behav. (2009) 96:18–22. doi: 10.1016/j.physbeh.2008.07.030, PMID: 18761027

[6] Fakhoury M. Role of the endocannabinoid system in the pathophysiology of schizophrenia. Mol Neurobiol. (2017) 54:768–78. doi: 10.1007/s12035-016-9697-5, PMID: 26768595

[7] Urban-Kowalczyk M Pigońska J Śmigielski J. Pain perception in schizophrenia: Influence of neuropeptides, cognitive disorders, and negative symptoms. Neuropsychiatr Dis Treat. (2015) 11:2023–31. doi: 10.2147/NDT.S87666, PMID: 26273205 PMC4532169

[8] Horvath G Liszli P Kekesi G Büki A Benedek G. Characterization of exploratory activity and learning ability of healthy and “schizophrenia-like” rats in a square corridor system (AMBITUS). Physiol Behav. (2017) 169:155–64. doi: 10.1016/j.physbeh.2016.11.039, PMID: 27923717

[9] Horvath G Liszli P Kekesi G Büki A Benedek G. Cognitive training improves the disturbed behavioral architecture of schizophrenia-like rats, “Wisket. Physiol Behav. (2019) 201:70–82. doi: 10.1016/j.physbeh.2018.12.011, PMID: 30576695

[10] Kekesi G Petrovszki Z Benedek G Horvath G. Sex-specific alterations in behavioral and cognitive functions in a “three hit” animal model of schizophrenia. Behav Brain Res. (2015) 284:85–93. doi: 10.1016/j.bbr.2015.02.015, PMID: 25698594

[11] Banki L Büki A Horvath G Kekesi G Kis G Somogyvári F. Distinct changes in chronic pain sensitivity and oxytocin receptor expression in a new rat model (Wisket) of schizophrenia. Neurosci Lett. (2020) 714:134561. doi: 10.1016/j.neulet.2019.134561, PMID: 31629032

[12] Horvath G Kis G Kekesi G Büki A Adlan LG Szűcs E. Interaction of clozapine with metformin in a schizophrenia rat model. Sci Rep. (2021) 11:16862. doi: 10.1038/s41598-021-96478-2, PMID: 34413440 PMC8376983

[13] Szűcs E Ducza E Büki A Kekesi G Benyhe S Horvath G. Characterization of dopamine D2 receptor binding, expression and signaling in different brain regions of control and schizophrenia-model Wisket rats. Brain Res. (2020) 1748:147074. doi: 10.1016/j.brainres.2020.147074, PMID: 32858029

[14] Holahan MR Rekart JL Sandoval J Routtenberg A. Spatial learning induces presynaptic structural remodeling in the hippocampal mossy fiber system of two rat strains. Hippocampus. (2006) 16:560–70. doi: 10.1002/hipo.20185, PMID: 16685708

[15] Kumar G Talpos J Steckler T. Strain-dependent effects on acquisition and reversal of visual and spatial tasks in a rat touchscreen battery of cognition. Physiol Behav. (2015) 144:26–36. doi: 10.1016/j.physbeh.2015.03.001, PMID: 25744936

[16] Peinado A Abrams CK. Patterns of spontaneous local network activity in developing cerebral cortex: relationship to adult cognitive function. PLoS One. (2015) 10:e0131259. doi: 10.1371/journal.pone.0131259, PMID: 26098958 PMC4476761

[17] Gogos A Sbisa A Witkamp D van den Buuse M. Sex differences in the effect of maternal immune activation on cognitive and psychosis-like behaviour in Long Evans rats. Eur J Neurosci. (2020) 52:2614–26. doi: 10.1111/ejn.14671, PMID: 31901174

[18] Uttl L Petrasek T Sengul H Svojanovska M Lobellova V Vales K. Chronic MK-801 application in adolescence and early adulthood: A spatial working memory deficit in adult long-evans rats but no changes in the hippocampal NMDA receptor subunits. Front Pharmacol. (2018) 9:42. doi: 10.3389/fphar.2018.00042, PMID: 29487522 PMC5816576

[19] Horvath G Plesz SB Ducza E Varga D Szucs E Benyhe S. Repurposing caffeine, metformin, and furosemide to target schizophrenia-related impairments in a triple-hit rat model. Int J Mol Sci. (2025) 26:6019. doi: 10.3390/ijms26136019, PMID: 40649798 PMC12249498

[20] Freund J Brandmaier AM Lewejohann L Kirste I Kritzler M Krüger A. Emergence of individuality in genetically identical mice. Sci (New York N.Y.). (2013) 340:756–9. doi: 10.1126/science.1235294, PMID: 23661762

[21] Kempermann G Lopes JB Zocher S Schilling S Ehret F Garthe A. The individuality paradigm: Automated longitudinal activity tracking of large cohorts of genetically identical mice in an enriched environment. Neurobiol Dis. (2022) 175:105916. doi: 10.1016/j.nbd.2022.105916, PMID: 36336243

[22] Tecott LH Nestler EJ. Neurobehavioral assessment in the information age. Nat Neurosci. (2004) 7:462–6. doi: 10.1038/nn1225, PMID: 15114359

[23] Castelhano-Carlos M Costa PS Russig H Sousa N. PhenoWorld: A new paradigm to screen rodent behavior. Trans Psychiatry. (2014) 4:e399. doi: 10.1038/tp.2014.40, PMID: 26126181 PMC4080321

[24] De Visser L Van Den Bos R Spruijt BM. Automated home cage observations as a tool to measure the effects of wheel running on cage floor locomotion. Behav Brain Res. (2005) 160:382–8. doi: 10.1016/j.bbr.2004.12.004, PMID: 15863235

[25] Kiryk A Janusz A Zglinicki B Turkes E Knapska E Konopka W. IntelliCage as a tool for measuring mouse behavior – 20 years perspective. Behav Brain Res. (2020) 388:112620. doi: 10.1016/j.bbr.2020.112620, PMID: 32302617

[26] Logan S Owen D Chen S Chen WJ Ungvari Z Farley J. Simultaneous assessment of cognitive function, circadian rhythm, and spontaneous activity in aging mice. GeroScience. (2018) 40:123–37. doi: 10.1007/S11357-018-0019-X, PMID: 29687240 PMC5964055

[27] Marwari S Dawe GS. Effects of haloperidol on cognitive function and behavioural flexibility in the IntelliCage social home cage environment. Behav Brain Res. (2019) 371:111976. doi: 10.1016/j.bbr.2019.111976, PMID: 31136773

[28] Zhang C Liu Q Yu CY Wang F Shao Y Sun KS. G protein-coupled estrogen receptor 1 knockout deteriorates MK-801-induced learning and memory impairment in mice. Front Behav Neurosci. (2020) 14:2020.00157. doi: 10.3389/fnbeh.2020.00157, PMID: 33324181 PMC7726131

[29] Hernadi Z Kormoczi L Adlan LG Kekesi G Büki A Liszli P. Categorized and individualized behavioral phenotyping approaches for control and triple-hit schizophrenia-like model rats in acute and chronic reward-based systems: A pilot study. PloS One. (2025) 20:e0328460. doi: 10.1371/journal.pone.0328460, PMID: 40773442 PMC12331104

[30] Leclerc MP Regenbogen C Hamilton RH Habel U. Some neuroanatomical insights to impulsive aggression in schizophrenia. Schizophr Res. (2018) 201:27–34. doi: 10.1016/j.schres.2018.06.016, PMID: 29908715

[31] Amitai N Markou A. Disruption of performance in the five-choice serial reaction time task induced by administration of N-methyl-D-aspartate receptor antagonists: Relevance to cognitive dysfunction in schizophrenia. Biol Psychiatry. (2010) 68:5–16. doi: 10.1016/j.biopsych.2010.03.004, PMID: 20488434 PMC2900523

[32] Evenden J. Impulsivity: A discussion of clinical and experimental findings. J Psychopharmacol. (1999) 13:180–92. doi: 10.1177/026988119901300211, PMID: 10475725

[33] Hoptman MJ. Impulsivity and aggression in schizophrenia: A neural circuitry perspective with implications for treatment. CNS Spectrums. (2015) 20:280–6. doi: 10.1017/S1092852915000206, PMID: 25900066 PMC4441843

[34] Bruinsma B Terra H de Kloet SF Luchicchi A Timmerman AJ Remmelink E. An automated home-cage-based 5-choice serial reaction time task for rapid assessment of attention and impulsivity in rats. Psychopharmacology. (2019) 236:2015–26. doi: 10.1007/s00213-019-05189-0, PMID: 30826849 PMC6647605

[35] Koot S Adriani W Saso L van den Bos R Laviola G. Home cage testing of delay discounting in rats. Behav Res Methods. (2009) 41:1169–76. doi: 10.3758/BRM.41.4.1169, PMID: 19897825

[36] Orduna V. Impulsivity and sensitivity to amount and delay of reinforcement in an animal model of ADHD. Behav Brain Res. (2015) 294:62–71. doi: 10.1016/j.bbr.2015.07.046, PMID: 26225844

[37] Remmelink E Chau U Smit AB Verhage M Loos M. A one-week 5-choice serial reaction time task to measure impulsivity and attention in adult and adolescent mice. Sci Rep. (2017) 7:42519. doi: 10.1038/srep42519, PMID: 28198416 PMC5309744

[38] Petrovszki Z Adam G Tuboly G Kekesi G Benedek G Keri S. Characterization of gene-environment interactions by behavioral profiling of selectively bred rats: The effect of NMDA receptor inhibition and social isolation. Behav Brain Res. (2013) 240:134–45. doi: 10.1016/j.bbr.2012.11.022, PMID: 23195116

[39] Ramzan I Wong BK Corcoran GB. Pain sensitivity in dietary-induced obese rats. Physiol Behav. (1993) 54:433–5. doi: 10.1016/0031-9384(93)90231-4, PMID: 8415933

[40] Castelhano-Carlos MJ Baumans V. The impact of light, noise, cage cleaning and in-house transport on welfare and stress of laboratory rats. Lab Anim. (2009) 43:311–27. doi: 10.1258/la.2009.0080098, PMID: 19505937

[41] Evenden JL Ryan CN. The pharmacology of impulsive behaviour in rats: The effects of drugs on response choice with varying delays of reinforcement. Psychopharmacology. (1996) 128:161–70. doi: 10.1007/s002130050121, PMID: 8956377

[42] Fouyssac M Puaud M Ducret E Marti-Prats L Vanhille N Ansquer S. Environment-dependent behavioral traits and experiential factors shape addiction vulnerability. Eur J Neurosci. (2021) 53:1794–808. doi: 10.1111/ejn.15087, PMID: 33332672

[43] Blumensohn R Ringler D Eli I. Pain perception in patients with schizophrenia. J Nervous Ment Dis. (2002) 190:481–3. doi: 10.1097/00005053-200207000-00011, PMID: 12142852

[44] Chen T-H Chen Y-J Huang T-S Hsiao M Lin C-C Liu Y-P. Does positive feeling lead to more impulsiveness? - Implication of previous rewarded experience on location-dependent motoric impulsivity. Chin J Physiol. (2021) 64:218–24. doi: 10.4103/cjp.cjp_63_21, PMID: 34708713

[45] Clemens LE Jansson EKH Portal E Riess O Nguyen HP. A behavioral comparison of the common laboratory rat strains Lister Hooded, Lewis, Fischer 344 and Wistar in an automated homecage system. Genes Brain Behav. (2014) 13:305–21. doi: 10.1111/gbb.12093, PMID: 24119005

[46] Godynyuk E Bluitt MN Tooley JR Kravitz AV Creed MC. An open-source, automated home-cage sipper device for monitoring liquid ingestive behavior in rodents. eNeuro. (2019) 6:ENEURO.0292-19.2019. doi: 10.1523/ENEURO.0292-19.2019, PMID: 31533961 PMC6787345

[47] Grieco F Bernstein BJ Biemans B Bikovski L Burnett CJ Cushman JD. Measuring behavior in the home cage: study design, applications, challenges, and perspectives. Front Behav Neurosci. (2021) 15:735387. doi: 10.3389/fnbeh.2021.735387, PMID: 34630052 PMC8498589

[48] Hånell A Marklund N Manahan-Vaughan D. Structured evaluation of rodent behavioral tests used in drug discovery research. Front Behav Neurosci. (2014) 8:252. doi: 10.3389/fnbeh.2014.00252, PMID: 25100962 PMC4106406

[49] Iman IN Yusof NAM Talib UN Ahmad NAZ Norazit A Kumar J. The intelliCage system: A review of its utility as a novel behavioral platform for a rodent model of substance use disorder. Front Behav Neurosci. (2021) 15:683780. doi: 10.3389/fnbeh.2021.683780, PMID: 34149373 PMC8211779

[50] Jhuang H Garrote E Yu X Khilnani V Poggio T Steele AD. Automated home-cage behavioural phenotyping of mice. Nat Commun. (2010) 1:68. doi: 10.1038/NCOMMS1064, PMID: 20842193

[51] Mingrone A Kaffman A Kaffman A. The promise of automated home-cage monitoring in improving translational utility of psychiatric research in rodents. Front Neurosci. (2020) 14:618593. doi: 10.3389/FNINS.2020.618593, PMID: 33390898 PMC7773806

[52] Voikar V Gaburro S. Three pillars of automated home-cage phenotyping of mice: novel findings, refinement, and reproducibility based on literature and experience. Front Behav Neurosci. (2020) 14:575434. doi: 10.3389/fnbeh.2020.575434, PMID: 33192366 PMC7662686

[53] Woodard CL Bolaños F Boyd JD Silasi G Murphy TH Raymond LA. An automated home-cage system to assess learning and performance of a skilled motor task in a mouse model of Huntington’s disease. eNeuro. (2017) 4:ENEURO.0141-17.2017. doi: 10.1523/ENEURO.0141-17.2017, PMID: 28929129 PMC5602104

[54] Amitai N Powell SB Young JW. Phencyclidine increased while isolation rearing did not affect progressive ratio responding in rats: Investigating potential models of amotivation in schizophrenia. Behav Brain Res. (2019) 364:413–22. doi: 10.1016/j.bbr.2017.11.026, PMID: 29175446

[55] Audet MC Goulet S Doré FY. Impaired social motivation and increased aggression in rats subchronically exposed to phencyclidine. Physiol Behav. (2009) 96:394–8. doi: 10.1016/j.physbeh.2008.11.002, PMID: 19046980

[56] Deehan GA Cain ME Kiefer SW. Differential rearing conditions alter operant responding for ethanol in outbred rats. Alcoholism Clin Exp Res. (2007) 31:1692–8. doi: 10.1111/j.1530-0277.2007.00466.x, PMID: 17651466

[57] McCool BA Chappell AM. Early social isolation in male Long-Evans rats alters both appetitive and consummatory behaviors expressed during operant ethanol self-administration. Alcoholism Clin Exp Res. (2009) 33:273–82. doi: 10.1111/j.1530-0277.2008.00830.x, PMID: 19032581 PMC2633417

[58] Winstanley CA. The utility of rat models of impulsivity in developing pharmacotherapies for impulse control disorders. Br J Pharmacol. (2011) 164:1301–21. doi: 10.1111/J.1476-5381.2011.01323.X, PMID: 21410459 PMC3229763

[59] Carr MR van Mourik Y Gómez-Sotres P Solinas M de Vries TJ Pattij T. Assessment of impulsivity using an automated, self-adjusting delay discounting procedure. Behav Brain Res. (2025) 480:115405. doi: 10.1016/j.bbr.2024.115405, PMID: 39706531

[60] Kempermann G. Environmental enrichment, new neurons and the neurobiology of individuality. Nat Rev Neurosci. (2019) 20:235–45. doi: 10.1038/s41583-019-0120-x, PMID: 30723309

[61] Carrillo-Roa T Labermaier C Weber P Herzog DP Lareau C Santarelli S. Common genes associated with antidepressant response in mouse and man identify key role of glucocorticoid receptor sensitivity. PLoS Biol. (2017) 15:e2002690. doi: 10.1371/journal.pbio.2002690, PMID: 29283992 PMC5746203

[62] Herzog DP Beckmann H Lieb K Ryu S Müller MB. Understanding and predicting antidepressant response: using animal models to move toward precision psychiatry. Front Psychiatry. (2018) 9:512. doi: 10.3389/fpsyt.2018.00512, PMID: 30405454 PMC6204461

[63] Insel TR Cuthbert BN. Medicine. Brain disorders? Precisely. Sci (New York N.Y.). (2015) 348:499–500. doi: 10.1126/science.aab2358, PMID: 25931539

[64] Krishnan V Han M-H Graham DL Berton O Renthal W Russo SJ. Molecular adaptations underlying susceptibility and resistance to social defeat in brain reward regions. Cell. (2007) 131:391–404. doi: 10.1016/j.cell.2007.09.018, PMID: 17956738

[65] Andrews JS Jansen JHM Linders S Princen A Broekkamp CLE. Performance of four different rat strains in the autoshaping, two-object discrimination, and swim maze tests of learning and memory. Physiol Behav. (1995) 57:785–90. doi: 10.1016/0031-9384(94)00336-X, PMID: 7777618

[66] Weiss IC Pryce CR Jongen-Rêlo AL Nanz-Bahr NI Feldon J. Effect of social isolation on stress-related behavioural and neuroendocrine state in the rat. Behav Brain Res. (2004) 152:279–95. doi: 10.1016/J.BBR.2003.10.015, PMID: 15196796

[67] Rivalan M Munawar H Fuchs A Winter Y. An automated, experimenter-free method for the standardised, operant cognitive testing of rats. PLoS One. (2017) 12:e0169476. doi: 10.1371/journal.pone.0169476, PMID: 28060883 PMC5218494

