# Correction: Behavioral characterization of triple-hit schizophrenia-like Lisket rats derived from the Long Evans strain through acute and chronic behavioral tests

**DOI:** 10.3389/fpsyt.2025.1733302

**Published:** 2025-11-03

**Authors:** Anna Zoldi, László Kormoczi, Szonja B. Plesz, Leatitia G. Adlan, Gabriella Kekesi, Péter Liszli, Laszló G. Nyúl, Gábor Braunitzer, Gyöngyi Horvath

**Affiliations:** ^1^ Department of Physiology, Albert Szent-Györgyi Medical School, University of Szeged, Szeged, Hungary; ^2^ Department of Image Processing and Computer Graphics, Faculty of Science and Informatics, University of Szeged, Szeged, Hungary; ^3^ Sztárai Institute, University of Tokaj, Sárospatak, Hungary

**Keywords:** impulsivity, homecage system, learning, Long Evans rats, schizophrenia

In the ‘*Results*’ section of the **Abstract**, it was stated that “Lisket rats exhibited significantly increased pain sensitivity, reduced locomotion and exploration, and impaired learning ability”.

This has been corrected to read:

“Lisket rats exhibited significantly decreased pain sensitivity, reduced locomotion and exploration, and impaired learning ability.”

The original version of this article has been updated.

## Publisher‘s note

All claims expressed in this article are solely those of the authors and do not necessarily represent those of their affiliated organizations, or those of the publisher, the editors and the reviewers. Any product that may be evaluated in this article, or claim that may be made by its manufacturer, is not guaranteed or endorsed by the publisher.

